# Pyrene fate affected by humic acid amendment in soil slurry systems

**DOI:** 10.1186/1754-1611-2-11

**Published:** 2008-09-10

**Authors:** Yanna Liang, Darwin L Sorensen, Joan E McLean, Ronald C Sims

**Affiliations:** 1 Department of Civil and Environmental Engineering, Southern Illinois University Carbondale, 1230 Lincoln Dr., Carbondale, Illinois, USA; 2 Utah Water Research Laboratory, Utah State University, 1600 Canyon Road, Logan, Utah, USA; 3 Department of Biological & Irrigation Engineering, Utah State University, 4105 Old Main Hill, Logan, Utah, USA

## Abstract

**Background:**

Humic acid (HA) has been found to affect the solubility, mineralization, and bound residue formation of polycyclic aromatic hydrocarbons (PAHs). However, most of the studies on the interaction between HA and PAH concentrated on one or two of the three phases. Few studies have provided a simple protocol to demonstrate the overall effects of HA on PAH distribution in soil systems for all three phases.

**Methods:**

In this study, three doses of standard Elliott soil HA (ESHA), 15, 187.5, and 1,875 μg ESHA/g soil slurry, were amended to soil slurry systems. ^14^C-pyrene was added to the systems along with non-radiolabeled pyrene; ^14^C and ^14^CO_2 _were monitored for each system for a period of 120 days.

**Results:**

The highest amendment dose significantly increased the ^14^C fraction in the aqueous phase within 24 h, but not after that time. Pyrene mineralization was significantly inhibited by the highest dose over the 120-day study. While organic solvent extractable ^14^C decreased with time in all systems, non-extractable or bound ^14^C was significantly enhanced with the highest dose of ESHA addition.

**Conclusion:**

Amendment of the highest dose of ESHA to pyrene contaminated soil was observed to have two major functions. The first was to mitigate CO_2 _production significantly by reducing ^14^CO_2 _from ^14^C pyrene mineralization. The second was to significantly increase stable bound ^14^C formation, which may serve as a remediation end point. Overall, this study demonstrated a practical approach for decontamination of PAH contaminated soil. This approach may be applicable to other organic contaminated environments where active bioremediation is taking place.

## Background

Bioremediation has been recognized as an effective approach for polycyclic aromatic hydrocarbon (PAH) contaminated sites. Major characteristics of PAHs are low water solubility and high hydrophobicity, which limits availability to microorganisms. In order to increase the bioavailability of PAHs in soil or sediment, humic substance (HS) addition has been considered to be a better choice than chemical surfactant that may cause the loading of soil with chemicals whose future behavior, toxicity, and degradability cannot be predicted [1].

As the most abundant pool of nonliving organic matter in the environment [2] and having a unique constellation of reactive features [3], HS has been studied for its effect on PAH bioremediation and the possibility of using it as a natural attenuating agent for cost effective, in situ bioremediation [4]. Three major effects have been identified. The first effect is on PAH solubility, with several studies showing increased apparent aqueous solubility [5-12]. The second effect is the enhancement of PAH mineralization and biodegradation [10,13-17]. However, some other studies have shown that HS has no effects on PAH biodegradation [18-24]. The third effect is that HS may bind PAHs and form bound residues. Some characteristics of bound residues are that they are not bioavailable for further degradation, are nontoxic, and can be an environmental bioremediation endpoint [25-28]. The humic acid (HA) fraction of HS has been identified as the primary sink for bound residues of pyrene [29]. Even though pyrene is regarded as a recalcitrant contaminant, its degradation products and metabolites have been isolated and identified in laboratory cultures, soil microcosms, and environmental samples [30-32]. Incorporation of metabolites into soil HA is also considered to be a major mechanism for bound residue formation [26,33].

The distribution of pyrene and its degradation products in soil occurs among three major phases including: (1) air (mineralized as CO_2_), (2) water, and (3) solid. Solid phase pyrene can be separated into two components: (1) organic solvent extractable pyrene, and (2) nonextractable or bound pyrene [29]. While most of the studies on the interaction between HA and PAH evaluated one or two of the three phases, few studies provide a simple protocol to determine the overall effects of HA on PAH distribution in soil systems in all three phases.

A soil sample from the Champion International Superfund Site located in Libby, Montana, was used in this study [34]. This site experienced extensive contamination from wood-treating operations from 1946–1969. In a previous study, Nieman et al. [29] demonstrated that 11% of the ^14^C added as radiolabeled pyrene was bound to the native soil organic matter (soil organic carbon of 1.4%) in biologically active soil microcosms compared to only 3% in poisoned controls. In the present study, standard Elliott soil humic acid (ESHA) and ^14^C pyrene mixed with non-radiolabeled pyrene were added to the Libby soil to increase bound residue formation and determine the formation pattern and stability of bound residue formed from newly added contaminants.

The objectives of this study were to: 1) present the distribution and mass balance of pyrene in soil slurry systems amended with ESHA, and 2) determine the short-term stability and degradability of bound residues formed.

## Methods

### Chemicals

Pyrene (99%) was purchased from Fluka (Buchs, Switzerland). Radio-labeled [4,5,9,10-^14^C] pyrene (95% purity, specific activity = 56 mCi/mmol) was purchased from Amersham International (Buckinghamshire, England). Analytical reagent grade sodium hydroxide (NaOH pellets) and potassium hydroxide (KOH pellets) were purchased from Mallinckrodt Baker Inc. (Paris, KY). Methanol and acetonitrile used were high-performance liquid chromatography (HPLC) grade or the equivalent. Ready Gel scintillation cocktail was bought from Beckman Coulter (Fullerton, CA). ESHA was purchased from International Humic Substance Society (IHSS) with a carbon content of 58.1%.

### Soil

The soil material used in this experiment was from the prepared bed land treatment unit 2 (LTU2) at the Champion International Superfund Site in Libby, MT. The soil had been contaminated by a mixture of creosote and pentachlorophenol used as a wood preservative at the site [34,35]. The soil sample was passed through a 2.0-mm sieve and homogenized. The homogenized soil was classified as a loam (50% sand, 38% silt, 12% clay) with an organic carbon content of 1.4%. Other physical and chemical properties of the soil sample were: pH, 7.6; potassium, 16 mg/l; NO_3_-N, <1.0 mg/kg; NaHCO_3 _extractable phosphorous, 13 mg/kg (analysis by Utah State University Soil Testing Laboratory). The soil was stored in the dark at 4°C until used. The moisture content was 10.2% (dry weight basis) immediately before use.

### ESHA effect on ^14^C mass balance

A previous publication has indicated that adding ESHA to soil at doses of 20–200 μg ESHA/g soil consistently increased pyrene mineralization by indigenous microorganisms in soil microcosms, whereas the lowest dose of 10 and other doses from 400 to 3,360 μg ESHA/g soil presented no effect and 10,080 μg ESHA/g soil produced inhibition [10]. Based on these dose effects, this study was conducted to evaluate how ESHA amendment affects ^14^C distribution among air, water, and solids. ESHA at doses of 15, 187.5, and 1,875 μg ESHA/g soil slurry was evaluated together with a control that had no ESHA addition. Duplicates of each treatment were analyzed at each sampling time of 1 h, 4 h, 16 h, 24 h, day 7, day 35, and day 120. A total of 56 microcosms were incubated.

The ESHA (30,000 mg/l) was dissolved in 0.1 M NaOH and the pH was adjusted to 7.0 using 4 M NaOH. Ten gram LTU2 soil (dry weight) in a 125-ml flask was spiked with ^14^C pyrene mixed with non-radiolabeled pyrene in methanol to make the final pyrene concentration of 100 mg/kg and the total disintegrations per minute (DPM) of 312,723 ± 692. After the methanol was evaporated in a fume hood, 30 ml of an aqueous solution consisting of either deionized distilled water (DDW) or DDW with different amounts of ESHA were added to each control and treatment, respectively. For the doses of 15, 187.5, and 1,875 μg ESHA/g soil slurry, the aqueous solutions contained 0.02 ml ESHA solution with 30 ml DDW, 0.25 ml ESHA solution with 29.75 ml DDW, and 2.5 ml ESHA solution with 27.5 ml DDW, respectively. Flasks were then transferred into clean, one-quart mason jars with Teflon coated lids. Carbon dioxide traps consisting of 2.5 ml of 0.1 M KOH in 20 ml scintillation vials were also included in each jar. All systems were incubated in the dark at 30°C on a rotary shaker at 105 rpm.

At each sampling time, ^14^CO_2 _traps were analyzed by liquid scintillation counting (LSC). The entire volume of the slurry in each flask was transferred to a pre-weighed 50-ml polypropylene centrifuge tube. After centrifuging at 2,000 × g for 30 min, the supernatant was transferred into another pre-weighed centrifuge tube. One ml of supernatant from each treatment and control was pipetted into a 7-ml scintillation vial with the addition of 6 ml Ready Gel scintillation cocktail and counted for aqueous phase ^14^C. Due to the dark brown color of the aqueous phase of the treatment with 1,875 μg ESHA/g soil slurry, 100 μl sample was analyzed as described above to avoid color interference with LSC counting.

The pellet was air-dried in a fume hood for three days. ^14^C in the solids was extracted by sonication (Tekmar Sonic Disruptor) twice with each extraction lasting 5 min on a full power, pulsed sonication cycle with 20 ml acetonitrile. The extraction efficiency was calculated as 90% using standard ^14^C pyrene. The acetonitrile extract was decanted and centrifuged for 40 min at 2,000 × g. The supernatant was transferred to a pre-weighed centrifuge tube and 200 μl of each solvent extract (SER) was placed in a 7-ml scintillation vial with 6 ml Ready Gel scintillation cocktail and counted by LSC.

The remaining soil sample was air-dried for three days and then ground in a mortar and pestle. A 0.5 g subsample was taken for combustion to determine non-extractable or bound residue (BR) ^14^C (Harvey Biological Oxidizer, RJ Harvey Instrument Corp., NJ). ^14^CO_2 _was trapped in a mixed solution of 50% ready gel, 40% methanol, and 10% monoethanolamine (MEA) and counted by LSC. Instrument recovery analysis was performed every 20 samples using ^14^C pyrene added to sand as a standard. The average standard recovery was 91%.

### Statistical analysis

JMP IN 5.1 statistical analysis software (SAS institute, NC) was used to analyze all experimental data. Experimental results from mineralized, aqueous, SER, and BR ^14^C were analyzed using a factorial design with time, treatments, and time * treatments as factors with the Fit Least Squares model. When the factors in the ANOVA and effect tests were determined to be significant (α = 0.05), multiple comparison analyses through LSMeans, Tukey's honest significant difference (HSD) were reported. The HSD was calculated and labeled on each graph, where needed.

## Results

For the three treatments and the control, the overall recovery of ^14^C added to the soil slurry systems was 100 ± 4% for the first 24 h, which indicated that the procedures used for analyzing the dissolved, bound, and extractable phases were valid. Recoveries from day 7 to day 120 ranged between 70% and 89%. The lower recovery at longer incubation times are likely due to CO_2 _emissions from the microcosms and/or low trapping efficiency for ^14^CO_2 _as this was also demonstrated in Nieman's [29] study. However, since the focus of this study was to evaluate the distribution of ^14^C once it was added to the soil sample, this low recovery would not be expected to effect soil BR formation and measurement.

**Figure 1:**
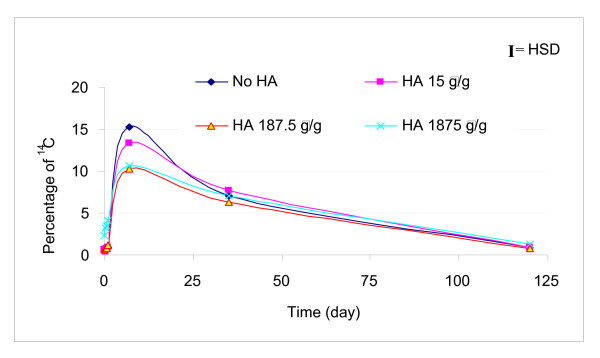
**Change in ^14^C in aqueous phase with time**. For the three treatments amended with standard Elliott soil humic acid (ESHA) and the control (no ESHA addition), aqueous phase ^14^C fraction increased with time to day 7 and then decreased with time.

**Figure 2:**
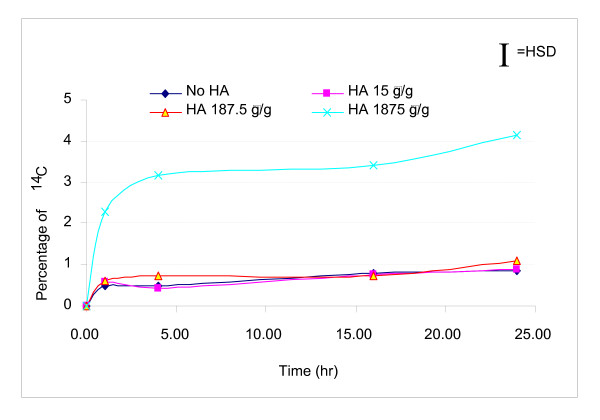
**During the first 24 h, percentages of ^14^C in aqueous phase change with time**. The largest dose of ESHA showed statistically significant enhancement of the aqueous phase ^14^C fraction compared to the other systems.

**Figure 3:**
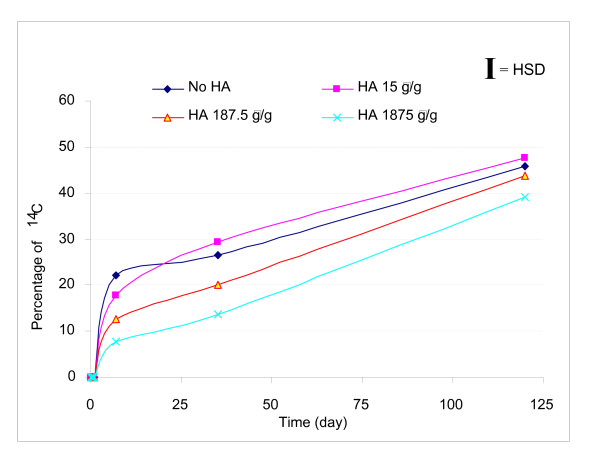
**Cumulative percentages of ^14^C pyrene as mineralized ^14^CO_2 _with time**. For all four systems, mineralization increased with time within the experimental period. Statistical analysis showed that the two higher doses of ESHA amendment significantly inhibited pyrene mineralization compared to the lowest dose ESHA amendment and the control over time.

The ^14^C in the aqueous phase increased over the first 7 days of incubation with the highest aqueous concentration in the control and 15 μg/g ESHA treatment (Figure 1). After 7 days, the aqueous concentration decreased for all systems. During the first 24 h, the highest dose of 1,875 μg ESHA/g soil slurry amendment was associated with a significant increase of ^14^C in the aqueous phase fraction compared to those of the control and the other two lower doses as shown in Figure 2. Mineralization was observed in the four systems by 24 h (Figure 3). Initially, the mineralization of pyrene was inhibited with ESHA dosing of 187.5 and 1,875 μg/g compared with the control and lowest ESHA dosing. By day 120, the mineralization at the highest dosing of ESHA was still less than the control.

**Figure 4:**
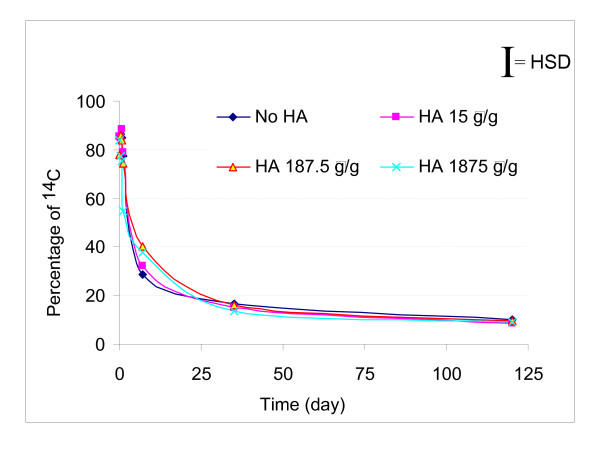
**Percentages of ^14^C as solvent extractable (SER) in the soil matrix change with time**. For all four systems, SER fraction decreased with time rapidly during the first 35 days and then slowly to day 120.

SER ^14^C decreased gradually from an average of 85% measured at 1 h of incubation to 8–10% on day 120 for the four systems (Figure 4). This experiment showed that after ^14^C pyrene was added to the soil, most of ^14^C was associated with the soil matrix and was apparently solvent extractable. When mineralization began at day 1, SER ^14^C underwent rapid mass transfer from the soil matrix to the aqueous phase and was degraded. A decrease of approximately 40% of SER ^14^C was observed between day 1 and day 7. By the end of the experiment, an average of 9% of the added ^14^C remained solvent extractable. Statistical analysis showed that there was no significant difference in SER ^14^C recovery among the four systems.

**Figure 5:**
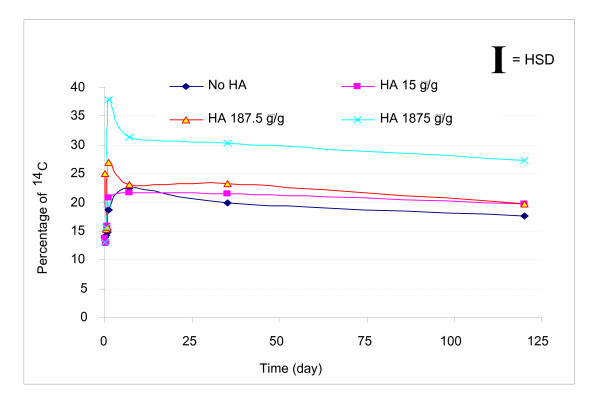
**Percentages of ^14^C as bound residue (BR) change with time**. BR fractions in the four systems increased with time during the first 24 h. For the two larger doses of ESHA amendment, 187.5 and 1,875 μg ESHA/g soil slurry, BR fractions decreased from day 1 to day 7 rapidly and then remained constant throughout the experimental period. The highest dose of ESHA addition increased the BR fraction significantly compared to other systems.

BR formation was observed to be a very rapid process (Figure 5). After ^14^C pyrene was added to the soil, approximately 13% of the ^14^C became non-extractable within 1 h. For the control and lowest ESHA dosing, the percentage of ^14^C BR remained constant at 14% over the first 16 h of incubation, then increased by day 7, and remained constant over the remaining time of the 120 day experiment.

For the two higher doses of ESHA amendment, percentages of BR ^14^C during the first 16 h were similar to those of the control and the lowest dose addition with an average of 15% ^14^C bound. The highest percentages of BR ^14^C were observed by day 1 with 27% and 38% of ^14^C bound for 187.5 and 1,875 μg ESHA/g soil slurry, respectively. The BR decreased by day 7 and remained constant thereafter. Statistical analysis indicated a significant difference between the BR fraction at the highest dose of ESHA amendment and the other three systems.

## Discussion

The SER ^14^C is the fraction that is associated with the soil matrix by reversible adsorption through a combination of van der Waals forces, hydrogen bonds, hydrophobic interactions, ionic bonds, ligand exchange, and charge transfer complexes [25] and can be extracted by organic solvents [36,37]. After ^14^C pyrene was added to the soil, it was sorbed to the soil matrix with approximately 85% as SER and 15% as BR for the four systems. When water and ESHA solution were added to the soil matrix, ^14^C distribution among air as ^14^CO_2_, water, and solid phases started to change differently for the different ESHA-dosed systems with time. Biodegradation acted as a driving force to pull SER ^14^C to the aqueous phase where the compound was mineralized. This is supported by the observation that the aqueous phase ^14^C fraction increased through day 7, then decreased to account for only 5% of the added ^14^C; but mineralization continued to increase for the rest of the time. During the 120-day experimental period, the control without ESHA amendment and the lowest dosing of 15 μg ESHA/g soil slurry were always statistically the same regarding ^14^C distribution among different physical phases of the soil slurry systems.

During the first 24 h, the highest dose of ESHA amendment significantly increased the aqueous phase pyrene fraction by a factor of five compared to those of the other systems. A similar phenomenon was observed in another study with the same ESHA and soil [10]. The role of HA in enhancing organic pollutant solubility has also been reported in two other studies, which indicated that HA could increase apparent solubility of trimethylnaphthalene, methylnaphthalene, and dimethylnaphthalene in aquifer systems [38], and natural organic matter could facilitate transport and enhance desorption of PAHs in aquifer sediments [8]. However, the time-dependent effect of this enhancement has not been discussed before. Moreover, even though the aqueous ^14^C fraction was increased in this experiment during the first 24 h with the highest dosing of ESHA, mineralization was not enhanced. This may indicate that the ESHA solubilized radiolabeled material was not bioavailable, possibly through micellar encapsulation, and/or that a community of microbes capable of appreciable mineralization of pyrene in this environment had not yet developed.

During the experimental period, mineralization in the soil slurry system with the highest dose of ESHA amendment was significantly lower compared to the other three systems. Similar conclusions were also drawn from studies by Lesage [9] and Spaccini [39]. These results indicate that a high dose of ESHA may inhibit pyrene mineralization or biodegradation by toxicity or by forming nonbioavailable micelles, which may precipitate or be pushed to the soil matrix by the hydrophobic effect. However, due to different HAs, different soil or sediment or soil slurry systems that have been tested by different researchers, it is difficult to identifya generalized dose for mineralization inhibition for different systems. To our best knowledge, results obtained in one system cannot be simply transferred to other different environments.

In these soil slurry systems, all three doses of ESHA amendment did not show enhancement of pyrene mineralization. In contrast, ESHA was reported to increase the pyrene degradation rate by *Mycobacterium *sp. JLS [15]. However, that observation was made in a non-slurry static system of ESHA and pyrene without the presence of soil and where the dose of ESHA was not known.

In this study, the BR fraction had four phases of change. First, the BR was formed immediately after pyrene was added to the soil. This spontaneous occurrence of bound residue formation was non-biologically derived as it was also observed in poisoned or sterile samples [25,40-42]. Second, the BR fraction increased with time by 24 h for the two higher doses of ESHA and by day 7 for the control and the lowest dosing of ESHA. A similar trend was reported when pyrene was added to municipal biowaste [43]. These two phases of change can be explained by a hydrophobic sorption mechanism proposed by Karickhoff [44] and Robinson et al. [45], which describes rapid hydrophobic interactions between PAHs and soil hydrophobic surfaces at the first step and a slow migration of PAHs to less accessible sites at the second step.

Third, the BR fraction decreased in the two higher doses of ESHA addition from 24 h to day 7 when mineralization was active. The decreased BR ^14^C fraction did not cause an increase of SER ^14^C, but it correlated well with increased aqueous phase ^14^C fraction and mineralization, which indicated that ^14^C released from the BR fraction was bioavailable and could be mineralized to ^14^CO_2 _as biodegradation became more aggressive [26,28,43,46]. Fourth, after day 7, the BR fraction in the four systems was constant.

BR formation and PAH retention were significantly increased by adding organic supplements to the soil in other studies [47-49]. However, addition of other supplements including mature compost, bark chips, or forest litter has not shown a positive effect on the BR formation [25]. In this experiment, while the two lower doses of 15 and 187.5 μg ESHA/g soil slurry had no significant difference compared to the control during the 120 day period, the largest dose of 1,875 μg ESHA/g soil slurry showed statistically significant enhancement of BR formation. By day 120, there was 10% more BR in the highest dose of ESHA amendment compared to that of the control. This is in agreement with the study of ^13^C labeled 2-decanol, where increased binding through hydrophobic protection by exogenous HA was reported [39].

BR formed during this experimental period was observed to be stable even though biodegradation was active. Therefore, amendment of HA-rich materials to soil slurry systems to increase BR formation could be considered as an effective treatment technology for the Libby Superfund site.

## Conclusion

The protocol developed in this study was effective to evaluate pyrene distribution among different physical phases of soil slurry systems, including air, water, and solid phases. Depending on the dose of EHSA, amending ESHA to soil slurries had significant effects on pyrene apparent solubility, mineralization, and BR formation. These effects can be applied as engineering management alternatives to achieve clean-up goals for PAH-contaminated sites.

## Competing interests

The authors declare that they have no competing interests.

## Authors' contributions

YL carried out the laboratory studies and drafted the manuscript. DLS has been involved in drafting the manuscript and revising it critically for important intellectual content. JEM participated in the statistical analysis and revised the manuscript critically for important intellectual content. RCS has been substantially involved in experimental design, data acquisition, analysis, and interpretation and revising the manuscript critically for important intellectual content. All authors read and approved the final manuscript.
